# Professor Ruth Guinsburg: her path and example leading the Revista Paulista de Pediatria

**DOI:** 10.1590/1984-0462/2025/43/Especial

**Published:** 2025-10-20

**Authors:** Fabio Carmona, Marina Carvalho de Moraes Barros, Túlio Konstantyner, Paloma Ferraz, Cléa Rodrigues Leone

**Affiliations:** 1 Universidade de São Paulo, Faculdade de Medicina de Ribeirão Preto – Ribeirão Preto (SP), Brazil.; 2 Universidade Federal de São Paulo, Escola Paulista de Medicina – São Paulo (SP), Brazil.; 3 Sociedade Paulista de Pediatria – São Paulo (SP), Brazil.; 4 Universidade de São Paulo, Faculdade de Medicina – São Paulo (SP), Brazil.

## THE LEGACY OF PROFESSOR RUTH GUINSBURG AT THE HELM OF RPPED

The trajectory of Professor Ruth Guinsburg's leadership at the *Revista Paulista de Pediatria* (RPPed) can be traced through a succession of objective milestones, the result of her visionary leadership and tireless dedication.

Her tenure as the editor-in-chief began in 2004. At that time, RPPed already held a solid regional tradition, yet had not achieved indexing in major international databases. One of her initial actions was the reorganization of the editorial board, incorporating researchers with established careers in various subspecialties of pediatrics.^1^

Over the following years, systematic improvements were introduced in the editorial workflow and author guidelines, with an explicit emphasis on encouraging the use of robust scientific methods and the presentation of reliable results that could practically contribute to the care of newborns, children, and adolescents. The adoption of an electronic submission system enhanced both the speed and transparency of the editorial process.^1^

In 2010, a major milestone was achieved with the inclusion of RPPed in the Scopus^®^ database, significantly increasing the journal's international visibility. Around the same time, the journal began publishing trilingual abstracts in Portuguese, English, and Spanish, thereby improving accessibility for readers across different countries.^1^

In 2013, RPPed achieved another notable milestone: inclusion in Medline/PubMed, one of the most respected medical literature databases worldwide. This recognition followed a rigorous international peer-review process and directly resulted from the editorial maturation fostered under Professor Guinsburg's leadership.^1^

In 2020, the journal took yet another significant step by being indexed in Web of Science, consolidating its presence among journals included in the Journal Citation Reports (JCR) databases. Consequently, RPPed became part of impact metric systems such as CiteScore and the impact factor, which are key indicators of quality and relevance in the global scientific publishing landscape. In 2023, RPPed adopted English as its sole publication language, thereby further expanding its international visibility.^1^

In 2025, at the conclusion of her tenure, Professor Guinsburg celebrated with joy and emotion one of the highest recognitions of her editorial journey. As communicated to the Editorial Board, scientometric data for 2024 confirmed that RPPed's CiteScore remained competitive at 2.2. Despite a slight decrease, consistent with a trend observed in most journals, the journal rose in Scopus's global pediatrics ranking, reaching the 53^rd^ percentile and entering the second quartile (Q2), becoming the second highest-ranked Latin American journal in the field.^2^

Even more remarkable was the performance in the JCR, where the journal's impact factor increased substantially from 1.4 in 2023 to 2.0 in 2024. This growth propelled RPPed up 31 positions in the international pediatrics journal ranking, from 100^th^ to 69^th^ place, also placing it within the second quartile of JCR.^2^ These results confirm that RPPed has not only solidified its national relevance but has also attained a prominent position in the global pediatric scientific publishing landscape.

While these numbers are undoubtedly significant, they represent only the visible facet of a body of work that was deeply human and collaborative. In her farewell, Professor Guinsburg made a point of acknowledging the contributions of the entire editorial and administrative team, dedicating the journal's achievements to the memory of colleagues who have left a lasting mark on its history, and generously placing her trust in the incoming editorial team.

## A LEADERSHIP GROUNDED IN SCIENCE, ETHICS, AND EDUCATION

Professor Guinsburg's leadership at RPPed was consistently guided by a profound commitment to professional training and the social responsibility of knowledge dissemination. Her clinical insight as a neonatologist, combined with her experience as a researcher and university professor, endowed her editorial role with a unique sensibility: She was able to discern the value of a manuscript not only through its methods but also through its applicability, originality, and potential to transform pediatric practice.

Professor Guinsburg also implemented an active policy of promoting national scientific output, offering space to emerging research groups, regional centers of excellence, scholars from related fields of child and adolescent health, and multicenter studies led by residents and early-career researchers. Through this initiative, RPPed published dozens of thematic issues, special editions, and supplements on public health, epidemiological surveillance, immunization, infant mortality, child development, and the prevention of avoidable conditions.

It is no coincidence that, under her leadership, the journal became a key resource for continuing medical education in Brazil. Numerous articles published during her tenure have become essential readings in medical residency programs, specialist board exams, and academic recruitment processes.

## FOSTERING A SCIENTIFIC COMMUNITY

Another noteworthy achievement of Professor Guinsburg's leadership was the strengthening of the relationship between the journal and its readership. Over the years, she cultivated a solid editorial community composed of authors, reviewers, and readers, who recognized RPPed as a space for open, qualified, and ethically grounded scientific dialog aimed at improving child and adolescent health.

She fostered regular dialog with the pool of reviewers and developed tools to standardize the peer-review process, such as review forms, referee guidelines, and editorial decision criteria, which collectively improved the quality of peer review, making it more transparent and educational without compromising respect for the opinions and individuality of each scientific reviewer or editorial board member.

Her leadership also reinforced the relationship between RPPed and the *Sociedade de Pediatria de São Paulo* (SPSP). The journal became one of SPSP's most prominent scientific products, distributed electronically and in print to thousands of pediatricians. Another major achievement, championed by Professor Guinsburg, was maintaining free access to the journal's content through the Scientific Electronic Library Online (SciELO) platform, thereby democratizing access to knowledge even in regions distant from major academic centers.

Professor Guinsburg's editorial legacy will continue to inspire the next generation of pediatricians, researchers, and editors. Her vision, values, and contributions are now embedded in the institutional fabric of RPPed. As we move forward, we carry her legacy with responsibility, gratitude, and renewed commitment to scientific excellence.

## Data Availability

The database that originated the article is available with the corresponding author.
